# Emotional Demand and Mental Health in Korean Employees

**DOI:** 10.3390/ijerph18147312

**Published:** 2021-07-08

**Authors:** Soon-Chan Kwon, Inah Kim, Yu-Mi Kim

**Affiliations:** 1 Department of Occupational and Environmental Medicine, College of Medicine, Soonchunhyang University, Cheonan 31151, Korea; sckwon@sch.ac.kr; 2 Department of Occupational and Environmental Medicine, College of Medicine, Hanyang University, Seoul 04673, Korea; inahkim@hanyang.ac.kr; 3 School of Public Health, Hanyang University, Seoul 04673, Korea; 4 Department of Preventive Medicine, College of Medicine, Hanyang University, Seoul 04673, Korea

**Keywords:** emotional labor, emotional demand, depression, worker, suicidal ideation

## Abstract

Background: Emotional demand (ED) at work is related to mental health in the general workforce, not just emotional workers. We investigated the relationships between ED and mental health outcomes, including distress, depressive symptoms (DS), experience of depression (DE), and suicidal ideation (SI) on the entire general workforce using nationally representative data. Methods: 5787 full-time employees were analyzed using cross-sectional design with the fourth Korean National Health and Nutrition Examination Survey (K-NHANES IV). Work-related psychosocial factors and mental health status were measured through face-to-face interviews. Multi-stage and stratified survey designs were considered in the analysis, and the mental health effects of ED were analyzed using multivariable logistic analysis. The Cochran–Armitage trend test was conducted to investigate increases in the relationship between the severity of ED and mental health outcomes. Results: The subjects comprised 3089 men and 2698 women. ED was reported by 36.7% of men and 39.3% of women. The estimated prevalence of distress was 27.5% in men and 34.6% in women. Adjusted odds ratios (ORs) of ED for distress were 2.62 (95% confidence interval (CI) = 2.10–3.28) for men and 2.57 (95% CI = 1.92–3.45) for women. DS was significantly related to ED (men: OR = 1.72, 95% CI = 1.18–2.50; women: OR = 1.91, 95% CI = 1.33–2.74). ED was also significant psychosocial risk factor for DE (men: OR = 1.88, 95% CI = 1.07–3.29; women: OR = 1.77, 95% CI = 1.15–2.75) and SI (men: OR = 1.64, 95% CI = 1.11–2.41; women: OR = 2.31, 95% CI = 1.63–3.28). Conclusions: ED was a risk factor for distress, DS, DE, and SI in the general workforce. Legal and social safety networks should be constructed for workers whose emotions may be hidden at work, as well as workers in emotion-related fields.

## 1. Introduction

The European Agency for Safety and Health (EU-OSHA) has noted that emerging psychosocial hazards include new forms of employment contracts and associated job insecurity, an aging workforce, work intensification, poor work–life balance, and high emotional demand (ED) at work [1]. Approximately 28% of EU workers report exposure to psychosocial hazards, and 14% of individuals with work-related health problems have reported stress, depression, or anxiety as their main health problem, after musculoskeletal problems [2]. Many studies have reported an association between job stress and common mental health disorders, such as mood and anxiety disorders [3].

In the European Working Conditions Survey 2010, 25% of EU workers regarded emotional demand (“Hiding feelings”) as a characteristic of their job content [4]. Emotional labor has become an important component of jobs [5]. Numerous jobs now include emotional expression as a major task [6,7]. Emotional labor was first defined by Hochschild in the context of service work as “the management of feeling to create a publicly observable facial and bodily display” [8]. Workers who perform emotional labor must emote according to work-specific rules, leading to discrepancies between the emotions expressed by workers and those they actually feel [9]. High ED is related to such problems as depression and anxiety, fatigue, psychological distress, long-term sickness absence, increased risk of musculoskeletal symptoms, increased risk of occupational accident injury, and increased risk of poor perceived health status [10,11,12,13,14,15].

In the Republic of Korea, 70% of individuals work in the service sector [16]. Consequently, a large number of workers must engage in emotional labor and conform to emotional rules. In fact, the number of workers who must manage or suppress their emotions is likely much greater than the number engaged in “emotional labor” in a strict sense. We also assume that the additional ED would be higher in Korea than in other developed countries due to Korea’s patriarchal and elders-first Confucian culture, which also causes gender or age discrimination [17,18,19].

Therefore, we aimed to study the health effects of ED in the workplace using nationally representative data of the working population. We investigated the relationships between ED and workers’ mental health, such as distress, depressive symptoms (DS), experience of depression (DE), or suicidal ideation (SI) on the entire general workforce in the Republic of Korea.

## 2. Materials and Methods

### 2.1. Subjects

This study used representative data obtained from the fourth Korean National Health and Nutrition Examination Survey (K-NHANES IV), which has been conducted by the Ministry of Health and Welfare (MHW) since 1998. A detailed description of the K-NHANES design and data collection has been published elsewhere [20]. Households were randomly selected for participation through stratified multi-stage probability sampling based on geographical area. Six hundred geographical sampling units were used in the K-NHANES IV. Questionnaires on job stress were implemented only during the survey period. The response rate was 78.3%. Of the 24,871 people who participated in the health interview survey (4594 in 2007, 9744 in 2008, and 10,533 in 2009), 5787 full-time employees (FTEs) were included in our study.

### 2.2. Measurement

#### 2.2.1. Health Behaviors

Information regarding sex, age, marital status, alcohol consumption, smoking status, and exercise regime was obtained through interviews. The smoking group included current smokers, and the non-smoking group comprised those who had never smoked or were former smokers. “Problem drinking” was defined as consuming ≥7 and ≥5 glasses of alcohol ≥2 times per week for men and women, respectively. Moderate physical activity was defined as physical exercise for ≥30 min ≥5 times per week that led to slight shortness of breath or in which somewhat greater effort was expended than in usual daily activities.

#### 2.2.2. Socioeconomic Status

Household income was classified as low, middle-low, middle-high, or high (I, II, III, and IV) using K-NHANES-provided quadrants. Education level was divided into those who had (i) only completed elementary school or less, (ii) completed middle school or less, (iii) graduated from high school or completed high school, and (iv) attended college or higher. Categories for marital status were married, divorced, and never married.

#### 2.2.3. Occupational Characteristics

Occupation was classified into white-collar (managers and professionals), pink-collar (clerks, service, and sales workers), and blue-collar (agriculture/fishery workers, craft/trades workers, machine operators and assemblers, and elementary manual workers). We classified working hours per week as less than 40 h, 40–48 h, or >48 h. Work times were classified as “usually daytime (from 6 AM to 6 PM)” or “including nighttime.”

#### 2.2.4. Work-Related Psychosocial Factors Including ED

“Time pressure” was measured using the statement, “I usually work with time pressure due to workload.” “Decision authority” was measured using the statement, “I have the authority and influence to decide working hours or processes.” “Esteem” was measured via the statement, “Considering my efforts and contribution, I am held in esteem in my workplace.” ED was measured using the statement, “I have to work while hiding my real feelings.” Responses were given according to a four-point Likert scale with options of “strongly disagree,” “disagree,” “agree,” and “strongly agree.” We then grouped these four categories into “agree” or “disagree” for further analysis. However, to test for increasing trends, we used categories of “no” for “disagree” or “strongly disagree”, “moderate” for “agree”, and “severe” for “strongly agree.”

#### 2.2.5. Mental Health

We defined “distress” as responding “very much” or “much” (from Likert responses of “feel very much,” “feel much,” “feel less,” or “feel rarely”) to “I feel stress in daily living.” DS was defined as an affirmative answer to “I have felt sad or hopeless for at least two continuous weeks during the last year.” DE referred to a current diagnosis of depression by a physician. SI was defined as an affirmative answer to “I have thought that I wanted to die at some point in the last year.”

### 2.3. Statistical Analysis

We used SAS software version 9.3 (SAS Institute, Cary, NC, USA), specifically the PROC SURVEY procedure, to estimate proportions and adjusted odds ratios (ORs) for multi-stage and stratified survey designs. We set the integrated survey weights according to the official analysis guidelines of the Korean Center for Disease Control and Prevention. We also used the Cochran–Armitage trend test to examine the presence of a dose–response relationship between the severity of ED and mental health outcomes.

We conducted multivariable logistic regression to calculate the ORs with 95% confidence intervals (CIs) of ED for distress, DS, DE, or SI, while adjusting for age, presently smoking, problem drinking, household income, education, marriage, occupation, weekly working hours, working times, and work-related psychosocial factors such as time pressure, low decision authority, or inappropriate esteem. We also assessed the increasing tendency in the strength of association between ED (weak, moderate, or severe) and mental health outcomes while adjusting for socio-demographic, occupational, and psychosocial factors.

## 3. Results

### 3.1. Characteristics of Subjects

The subjects comprised 3089 men and 2698 women. The most common age groups were 30s (30–39 years) for men (32.9%) and 20s (19–29 years) for women (28.6%). The estimated smoking prevalence was 77.0% for men and 10.9% for women. The estimated prevalence of problem drinking was 28.1% for men and 8.8% for women. With regard to occupation, 39.7% were blue-collar workers, 42.1% were white-collar workers, and 18.2% were pink-collar workers. With regard to work, 16.8% of women and 7.1% of men reported less than 40 h of work per week, while 48.8% and 30.4%, respectively, worked more than 48 h per week. Night shift work was reported by 23.3% of men and 21.7% of women.

ED was reported by 36.7% of men and 39.3% of women. The estimated prevalence of distress was 27.5% in men and 34.6% in women. The prevalence of DS, DE, and SI for men was 7.9%, 6.3%, and 8.2%, respectively, and the estimated prevalence rates for women were higher than those for men, at 17.0%, 18.1%, and 19.4%, respectively (Table 1).

### 3.2. Relationships between Covariates and Mental Health Outcomes

Table 2 shows the relationships between socio-demographic characteristics, work-related psychosocial factors, or ED and mental health outcomes such as distress, DS, DE, or SI. The estimated prevalence of distress was lower in blue-collar workers (22.4% for men and 29.7% for women) than in white-collar workers (32.3% and 37.7%, respectively) and pink-collar workers (32.3% and 35.5%, respectively). Furthermore, the estimated prevalence of distress was higher among workers with long working hours, high time pressure, inappropriate esteem, or ED than among others. Men with low decision authority had lower distress (22.9%) than did those with high decision authority (29.1%), but the result for women was reversed. The estimated prevalence of DS was higher in blue-collar workers (10.0% for men and 20.9% for women) than in pink-collar workers (7.5% for men and 18.1% for women) and white-collar workers (5.8% for men and 13.5% for women). Time pressure, low decision authority, inappropriate esteem, and high ED were related to a high prevalence of DS. The estimated prevalence of DE was highest among blue-collar workers for men (5.5%) and pink-collar workers for women (19.7%). Time pressure, low decision authority, inappropriate esteem, and ED were all related to a high prevalence of DE. For both sexes, the prevalence of SI during the previous year was greater for older individuals (60 years and older) and those with low household income. The prevalence of SI was also higher among unmarried men (10.4%) than among married men (7.4%), but lower among unmarried women (19.2%) than among married women (19.7%). SI also had a higher prevalence among blue-collar workers (11.2% for men and 24.5% for women) and workers with work arrangements that included a night shift (9.8% for men and 22.8% for women). Time pressure, low decision authority, inappropriate esteem, and ED were related to a higher prevalence of SI in both sexes.

### 3.3. ORs of ED in Multivariable Models

Table 3 shows the ORs of ED in the multivariable models. ORs of ED for destress were 2.62 (95% CI = 2.10–3.28) for men and 2.57 (95% CI = 1.92–3.45) for women. DS was significantly related to ED (men: OR = 1.72, 95% CI = 1.18–2.50; women: OR = 1.91, 95% CI = 1.33–2.74). ED was also a significant psychosocial risk factor for DE (men: OR = 1.88, 95% CI = 1.07–3.29; women: OR = 1.77, 95% CI = 1.15–2.75) and SI (men: OR = 1.64, 95% CI = 1.11–2.41; women: OR = 2.31, 95% CI = 1.63–3.28).

### 3.4. The Trends of Strength of Association between ED and Mental Health Outcomes

**Figure 1:**
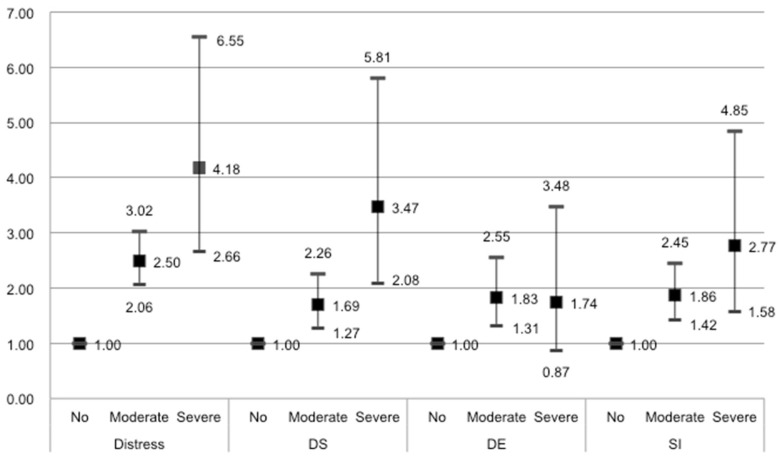
Trends in strength of association between ED and mental health outcomes (ORs and 95%CI). Adjusted for age, presently smoking, problem drinking, household income, education, marriage, occupation, weekly working hours, working times, time pressure, decision authority, and appropriate esteem. All P value were under 0.05 by the Cochran–Armitage trend test except DE. OR: odds ratio; ED: emotional demand; DS: depressive symptoms; DE: experience of depression; SI: suicidal ideation.

Figure 1 shows the results of the multivariable logistic regression indicating the trends in the strength of the association between ED and mental health outcomes after adjusting for socio-demographic, occupational, and psychosocial factors. We identified positive trends for the strength of the association between ED and distress (moderate ED: OR = 2.5, 95% CI = 2.1–3.0; severe ED: OR = 4.2, 95% CI = 2.7–6.6), DS (moderate: OR = 1.7, 95% CI = 1.3–2.3; severe: OR = 3.5, 95% CI = 2.1–5.8), and SI (moderate: OR = 1.9, 95% CI = 1.4–2.5; severe: OR = 2.8, 95% CI = 1.6–4.8), and *p* value by the Cochran–Armitage trend test were under 0.05. However, there was no statistically significant trend for DE (moderate: OR = 1.8, 95% CI = 1.3–2.6; severe: OR = 1.7, 95% CI = 0.9–3.5).

## 4. Discussion

This study showed that ED has a positive association with distress, DS, DE, and SI, and that the strength of associations for distress, DS, and SI increased with the severity of ED after adjusting for other covariates. This accords with the results of studies in the general Norwegian working-age population [21] and in a Dutch cohort of workers [13], wherein high ED was found to be related to psychological distress. A similar study in France found that “emotional demand”, defined as “hiding feelings at work” (which is congruent with our definition of ED), was related to major depressive disorder [22]. High ED has also been identified as a risk factor for depression among Danish workers [11], while emotional suppression is a risk factor for depression and anxiety among Korean workers. The ORs for the mental health problems of depression and anxiety disorder increased with the extent to which workers had to suppress emotion [23] and was a risk factor for SI among Korean service or sales workers, and the strength of this association increased when using an interaction model incorporating low job control [24].

As there is a limit to direct comparisons due to the differences in measurement methods across studies, the prevalence of mental health issues in the current study was higher than in other studies. The point prevalence of distress was higher (30.3%) in our study than was the prevalence of distress during the prior month for the Norwegian working-age population (16%) [21], and for Dutch workers (21%) [13]. The one-year prevalence of DS in Korean workers was similar to the lifetime prevalence of DE (respectively, 7.9% and 6.3% for men, and 17.0% and 18.1% for women). The values for women were two to three times higher than those for men. The prevalence of major depressive disorder in an interview survey of French workers was 3.8% for men and 7.9% for women [22], 4.0% for male and 9.0% for female American workers [25], and 3% for men and 6% for women in Canada [26]; notably, these values are all lower than those of Korean workers. According to the Center for Epidemiological Studies-Depression Scale (CES-D), the prevalence of DS in a Japanese tax office was 19.8% for men and 8.9% for women, which was similar to the prevalence in Korea [27]. Furthermore, the SI prevalence within the past year was 12.5%, which is higher than the 4.3% in French workers [28], 3.5% in Japanese middle-sized enterprise workers [29], 4.73% in Spanish workers [30], and 5.2% in Greek workers [31]. The higher prevalence of SI than DS and DE could be explained by the different recall period of each questionnaire for mental health outcomes.

This study found a relationship between ED and mental health outcomes after considering several covariates, but we could not identify the variables in which ED was involved in the pathway and/or mechanism leading to destress, DS, DE, and SI. In the relationship between ED and mental health outcomes, the indirect effects of anger suppression and anger rumination have been reported [32], and the work environment interactions, such as emotional display rules, angry customers, and health and safety information, have also been reported [33]. Burnout, a known consequence of ED, showed a negative relationship with emotional intelligence [34]. In order to elucidate the mechanisms and mediators that might cause mental health outcomes, studies of more robust design are needed.

Emotional dissonance between expressed and actual emotions is likely the main factor underlying the negative effects on mental health of hiding feelings at work. This surface acting results in negative effects on personal well-being, such as emotional exhaustion, depersonalization, psychological strain, and psychosomatic complaints, and on job-related well-being, such as job satisfaction and organizational attachment through emotion–rule dissonance (i.e., conflict between experienced and expressed emotions) according to the results of a meta-analysis. Expressing verbal emotions has positive effects on job performance, such as emotional performance and reported customer satisfaction [35].

As mentioned in the Background section, in the Republic of Korea, emotional laborers are usually women, and the intensity of ED could be higher than in other developed countries due to job quality and cultural context. In particular, patriarchal culture, which facilitates gender and age discrimination, might increase the intensity of ED—that is, hiding feelings at work may be continuously demanded of women, by both their family and workplace. The severe gender discrimination in the labor market with respect to income, job security, work separation, and decision authority likely also increases the intensity of ED for women and worsens their psychological stress [36].

Notably, we found an increasing strength in the association between ED and negative aspects of mental health, excluding DE, after adjusting for psychosocial risk factors. DE was defined on the basis of a past diagnosis by a doctor, so reverse causation is possible; that is workers with severe depression would likely be excluded from being FTEs in the labor market and the strength of the dose–response association could be weakened among those with severe ED. Although the times at which distress, DS, or SI occurred differed, and reverse causation must be considered, the results revealed consistently positive associations. Even if such associations originated from reverse causation, this would indicate that workers with mental health problems experience ED and psychosocial stress related to it.

This study had several limitations. First, although a representative sample of the Republic of Korea was analyzed, this study was cross-sectional. Consequently, we could not determine causal association. Although a longitudinal study has shown that workplace emotional needs are related to mental illness, including depression [37], most studies on this topic are cross-sectional. Second, this study used only simple questionnaire to measure ED and mental health outcomes. However, all the ORs of ED for the four mental health outcomes showed consistently positive associations. Furthermore, the ORs for mental health outcomes increased with increasing of ED intensity. These consistent results suggested that the measures of ED and mental health outcomes were reliable. “Hiding feelings at work” was also the most important question, and the same question was used in a previous French study [22]. Third, there could be potential confounders, although we presented the results adjusted for socio-demographics variables and work-related factors. Finally, we analyzed the K-NHANES Ⅳ (2007–2009), which seems outdated. Although we chose the data due to the national representative survey of the key variables, these were investigated only at that period; attention is needed to interpret and apply the findings.

## 5. Conclusions

In summary, this study found that ED was an important psychosocial risk factor for the mental health outcomes such as distress, DS, DE, and SI in both sexes, even after adjusting for other covariates among the general working population using nationally representative data. Policymakers should consider extending regulations to encompass all workers in order to prevent mental health problems, especially in the Korean context. Regulations to protect workers and promote mental health must cover not only service workers, but all workers in a range of industries.

## Figures and Tables

**Table 1:** General characteristics of study subjects, distributions of work-related psychosocial factors, and the estimated prevalence of mental health outcomes.

Variables		Men (*n* = 3089)		Women (*n* = 2698)		Total (*n* = 5787)	
		%	SE	%	SE	%	SE
Age	<19	1.4	0.24	1.5	0.28	1.4	0.19
	19–29	21.9	1.21	28.6	1.23	24.5	0.94
	30–39	32.9	1.28	22.9	0.98	29.0	0.99
	40–49	24.2	0.98	25.6	1.15	24.7	0.79
	50–59	14.8	0.76	14.3	0.80	14.6	0.58
	≥60	4.8	0.36	7.2	0.59	5.7	0.32
Presently smoking		77.0	1.00	10.9	0.80	51.4	0.82
Problem drinking		28.1	1.01	8.8	0.82	21.4	0.75
Moderate physical activity	No	86.9	0.76	86.6	0.83	86.8	0.60
Household income	I (lowest)	7.4	0.65	13.0	0.89	9.5	0.64
	II	23.1	1.17	27.2	1.19	24.7	0.99
	III	33.2	1.22	29.1	1.16	31.6	0.96
	IV (highest)	36.4	1.52	30.7	1.35	34.2	1.29
Education	Elementary	7.2	0.53	15.5	0.88	10.4	0.53
	Middle	8.2	0.68	11.0	0.71	9.3	0.56
	High	41.6	1.27	39.7	1.19	40.9	0.93
	College	43.0	1.41	33.8	1.20	39.4	1.12
Marriage	Yes	75.2	1.23	71.3	1.27	73.7	0.95
Occupation	Blue	45.3	1.35	30.9	1.21	39.7	1.06
	White	41.7	1.27	42.9	1.31	42.1	1.04
	Pink	13.0	0.77	26.2	1.13	18.2	0.65
Weekly working hours	<40	7.1	0.58	16.8	1.07	10.4	0.56
	40–48	44.0	1.19	52.8	1.46	47.0	0.93
	>48	48.8	1.21	30.4	1.42	42.5	0.95
Working times	Usually daytime	76.7	1.14	80.8	1.06	78.3	0.82
	Including nighttime	23.3	1.14	19.2	1.06	21.7	0.82
Time pressure		37.7	1.18	36.3	1.11	37.2	0.85
Low decision authority		26.7	1.00	39.6	1.13	31.7	0.78
Inappropriate esteem		11.6	0.72	10.7	0.83	11.2	0.58
Emotional demand		36.7	1.01	39.3	1.13	37.7	0.72
Distress		27.5	1.02	34.6	1.16	30.3	0.78
Symptoms of depression		7.9	0.57	17.0	0.83	11.4	0.51
Depression experience		6.3	0.47	18.1	0.66	13.1	0.46
Suicide ideation		8.2	0.62	19.4	0.89	12.5	0.58

*n*: number, SE: standard error.

**Table 2:** Sex stratified estimated prevalence of distress, depressive symptoms, depression experience, and suicide ideation by socio-demographic characteristics, work-related psychosocial factors, and emotional demands.

Variables	Categories	Distress		Depressive Symptoms		Depression Experience		Suicide Ideation	
		M	W	M	W	M	W	M	W
		%	%	%	%	%	%	%	%
Age	19–29	24.6	42.8	7.1	15.5	4.0	12.1	7.8	18.1
	30–39	35.0	33.7	7.4	15.0	2.0	13.7	7.6	16.5
	40–49	24.9	29.5	8.2	15.4	4.1	18.4	7.7	18.3
	50–59	24.7	31.7	9.3	23.4	4.5	18.8	10.5	22.5
	≥60	11.7	29.5	9.1	23.0	6.3	15.0	10.4	30.9
Present smoking	No	22.6	33.1	7.4	15.9	3.4	14.3	6.4	17.9
	Yes	29.0	46.4	8.0	25.7	3.5	22.9	8.8	31.4
Problem drinking	No	28.1	36.1	7.7	17.1	3.0	16.5	7.6	19.4
	Yes	28.1	43.9	7.9	22.3	3.3	16.3	8.6	29.9
Moderate physical activity	No	27.9	34.5	7.2	16.2	3.5	14.9	7.8	18.9
	Yes	24.5	35.6	12.4	22.6	4.2	18.4	10.9	22.8
Household income	I	26.2	38.2	11.7	25.4	10.4	17.2	15.3	29.5
	II	28.7	38.7	8.9	16.5	4.7	21.9	11.1	23.9
	III	28.2	33.3	7.3	17.4	2.3	15.0	7.0	18.4
	IV	26.9	31.6	6.7	13.9	2.4	9.2	5.6	11.9
Education	Elementary	23.8	30.6	14.5	24.3	11.0	15.2	21.3	27.5
	Middle	22.1	31.8	9.0	20.8	5.4	22.8	8.6	23.9
	High	27.0	32.8	8.6	16.3	3.1	16.0	9.4	18.9
	College	29.6	39.5	5.9	13.5	2.4	12.3	4.9	14.9
Marriage	No	22.9	41.3	8.1	16.6	4.5	11.7	10.4	19.7
	Yes	28.9	32.0	7.7	17.1	3.1	16.7	7.4	19.2
Occupation	Blue	22.4	29.7	10.0	20.9	5.5	17.4	11.2	24.5
	White	32.3	37.7	5.8	13.5	2.2	11.1	4.9	14.1
	Pink	32.3	35.5	7.5	18.1	2.0	19.7	9.5	21.7
Weekly working hours	<40	19.8	28.3	9.5	16.2	6.4	19.4	10.4	21.0
	40–48	24.7	32.0	6.2	13.2	2.4	10.9	5.9	14.9
	>48	33.1	44.6	8.5	19.7	3.5	16.6	9.5	22.1
Working times	Usually daytime	28.3	34.3	7.9	16.4	3.3	15.5	7.8	18.6
	Including nighttime	25.2	36.2	7.9	20.0	4.5	14.8	9.8	22.8
Time pressure	No	17.7	27.6	7.2	15.1	3.4	14.5	7.2	17.4
	Yes	43.5	46.7	9.1	20.5	3.8	16.8	10.0	22.8
Low decision authority	No	29.1	34.0	7.7	16.4	3.0	15.1	7.5	17.7
	Yes	22.9	35.7	8.2	18.0	5.2	15.7	10.1	22.1
Inappropriate esteem	No	25.6	33.0	7.6	15.7	3.4	14.3	7.7	18.1
	Yes	41.1	48.9	10.1	28.0	4.8	24.3	12.1	30.3
Emotional demand	No	19.3	25.6	6.6	13.1	2.5	12.7	6.6	14.3
	Yes	41.5	48.6	10.1	23.1	5.4	19.4	10.9	27.2

M: Men, W: Women, weight% were presented considering the multi-stage and stratified survey design.

**Table 3:** ORs of emotional demand for distress, depressive symptoms, depression experience, and suicide ideation.

Emotional Demand	Sex	Distress aOR (95% CI)		Depressive Symptoms aOR (95% CI)		Depression Experience aOR (95% CI)		Suicide Ideation aOR (95% CI)	
	Men	2.62	(2.10–3.28)	1.72	(1.18–2.50)	1.88	(1.07–3.29)	1.64	(1.11–2.41)
	Women	2.57	(1.92–3.45)	1.91	(1.33–2.74)	1.77	(1.15–2.75)	2.31	(1.63–3.28)

aOR: adjusted odds ratio; CI: confidence interval. Adjusted for age, presently smoking, problem drinking, household income, education, marriage, occupation, weekly working hours, working times, time pressure, decision authority, and appropriate esteem.

## Data Availability

The data that support the findings of this study are available from Korea Disease Control and Prevention Agency. https://knhanes.kdca.go.kr/knhanes/sub03/sub03_02_05.do.
